# VE-cadherin facilitates BMP-induced endothelial cell permeability and signaling

**DOI:** 10.1242/jcs.179960

**Published:** 2016-01-01

**Authors:** Andreas Benn, Clara Bredow, Isabel Casanova, Slobodan Vukičević, Petra Knaus

**Affiliations:** 1 Institute for Chemistry and Biochemistry, Freie Universität Berlin, Berlin 14195, Germany; 2 DFG Graduate School 1093 Berlin School of Integrative Oncology, Berlin 13353, Germany; 3 DFG Graduate School 203 Berlin-Brandenburg School for Regenerative Therapies, Berlin 13353, Germany; 4 Center for Translational and Clinical Research, University of Zagreb, Zagreb 10000, Croatia

**Keywords:** ALK2, ACVR1, BMP, BMPR2, Endothelial cells, Src, Permeability, VE-cadherin, Cadherin 5

## Abstract

Several vascular disorders, such as aberrant angiogenesis, atherosclerosis and pulmonary hypertension, have been linked to dysfunctional BMP signaling. Vascular hyperpermeability via distortion of endothelial cell adherens junctions is a common feature of these diseases, but the role of BMPs in this process has not been investigated. BMP signaling is initiated by binding of ligand to, and activation of, BMP type I (BMPRI) and type II (BMPRII) receptors. Internalization of VE-cadherin as well as c-Src kinase-dependent phosphorylation have been implicated in the loosening of cell–cell contacts, thereby modulating vascular permeability. Here we demonstrate that BMP6 induces hyperpermeabilization of human endothelial cells by inducing internalization and c-Src-dependent phosphorylation of VE-cadherin. Furthermore, we show BMP-dependent physical interaction of VE-cadherin with the BMP receptor ALK2 (BMPRI) and BMPRII, resulting in stabilization of the BMP receptor complex and, thereby, the support of BMP6-Smad signaling. Our results provide first insights into the molecular mechanism of BMP-induced vascular permeability, a hallmark of various vascular diseases, and provide the basis for further investigations of BMPs as regulators of vascular integrity, both under physiological and pathophysiological conditions.

## Abstract (precis)

**Summary:** We reveal the molecular mechanism by which BMP6 induces hyperpermeabilization of the endothelium. This provides first insights into the mechanism of BMP-dependent vascular integrity in normal physiology and disease.

## INTRODUCTION

Endothelial cells line the lumen of blood vessels and form a semi-permeable monolayer that controls blood–tissue exchange of fluids, plasma proteins and cells. Thus, the integrity of the endothelial barrier is essential for physiological tissue homeostasis, and hyperpermeability is involved in the progression of several pathological conditions, including inflammation, atherosclerosis and cancer (Davignon and Ganz, 2004; Weis and Cheresh, 2005).

Vascular permeability is mainly regulated by two mechanisms: transcellular and paracellular permeability. During transcytosis, macromolecules pass individual endothelial cells via specialized vesicles containing caveolin-1 (Bauer et al., 2005) or transmembrane channels formed by fused vesiculo-vacuolar organelles (VVOs) (Dvorak et al., 1996). Paracellular permeability is mediated by the dynamic opening of interendothelial cell junctions that allow large proteins and cells to enter the surrounding tissue. Alterations of cell–cell adhesion, mainly mediated by tight (TJs) and adherens (AJs) junctions, is a hallmark of paracellular permeability (Goddard and Iruela-Arispe, 2013). TJs mediate intercellular adhesion by the formation of complexes between several members of the claudin, occludin and junctional adhesion molecule (JAM) protein families and have been implicated in the regulation of the blood–brain barrier (Luissint et al., 2012). Vascular hyperpermeability in the microvasculature has been repeatedly linked to changes in AJ architecture. AJs are mainly formed by calcium-dependent interactions of vascular endothelial cadherins (VE-cadherins, also known as cadherin 5 or CD144) between neighboring cells. VE-cadherin is a single-span transmembrane protein exclusively expressed by endothelial cells and its extracellular domain forms homomeric dimers with VE-cadherin molecules of adjacent cells (Bravi et al., 2014). The cytoplasmic domain of VE-cadherin interacts with members of the actin-binding catenin family and thus links VE-cadherin to the cytoskeleton (Lampugnani et al., 1995; Iyer et al., 2004). VE-cadherin is the main component of endothelial AJs and homophilic VE-cadherin interactions have been reported to regulate vascular permeability. Among these regulatory mechanisms, internalization and tyrosine phosphorylation of VE-cadherin are strongly associated with impaired barrier function. It has been shown that vascular endothelial growth factor (VEGF, also known as VEGFA), a potent permeability-inducing agent, triggers VE-cadherin internalization (Gavard and Gutkind, 2006). Internalized VE-cadherin enters endosomal compartments and is either recycled to the plasma membrane or degraded (Xiao et al., 2003a, 2005). Hence, the promotion of VE-cadherin internalization controls permeability in a reversible manner. In addition, several studies have demonstrated that other permeabilizing agents, such as tumor necrosis factor α (TNF-α) (Angelini et al., 2006) and histamine (Andriopoulou et al., 1999), modulate AJ stability by tyrosine phosphorylation of VE-cadherin. It was shown that members of the Src kinase family phosphorylate VE-cadherin (Eliceiri et al., 1999) and Tyr685 of VE-cadherin was identified as a unique target site for c-Src *in vitro* (Wallez et al., 2007). Recently, it has been reported that phosphorylation at Tyr685 controls vascular permeability *in vivo*, while phosphorylation at Tyr731 regulates leukocyte diapedesis (Wessel et al., 2014). This highlights that modifications of VE-cadherin allow diverse alterations of AJ architecture and thereby dynamic disruptions of the endothelial cell barrier. This diversity is necessary considering that plasma protein and cell extravasation require openings of different sizes.

Besides controlling endothelial barrier function, VE-cadherin has been implicated in the regulation of growth factor signaling. VE-cadherin interacts with VEGFR2 (also known as KDR), and VE-cadherin clusters retain VEGFR2 at the plasma membrane (Lampugnani et al., 2006). Moreover, VE-cadherin has been implicated in the regulation of transforming growth factor β (TGF-β)-mediated inhibition of endothelial cell proliferation and migration (Rudini et al., 2008). It has been shown that VE-cadherin interacts with the TGF-β type I receptors activin receptor-like kinase 1 and 5 (ALK1 and ALK5, also known as ACVRL1 and TGFβR1, respectively), as well as the type II receptor (TGFβRII, or TGFβR2) and functions as a positive regulator of TGF-β signal transduction.

Bone morphogenetic proteins (BMPs) belong to the TGF-β family of secreted growth factors, which comprises more than 30 structurally related ligands that are tissue-specifically expressed and regulated by natural antagonists. BMPs bind to transmembrane serine/threonine kinase receptors and the signal is transduced into the cytosol via a heteromeric receptor complex of type I and type II receptors. Receptor complex activation results in induction of distinct intracellular signaling cascades: the canonical Smad and non-Smad pathways. Once receptor-regulated Smads (R-Smads, SMAD1/5/8) are activated upon phosphorylation by a type I receptor, they form a complex with the common mediator Smad (co-Smad, SMAD4) to function as transcription factors and, together with transcriptional co-activators and co-repressors, regulate the expression of BMP target genes such as *ID1* (Sieber et al., 2009).

BMP signal transduction is implicated in the regulation of physiological as well as pathological processes of the endothelium. Interestingly, altered BMP signaling has been observed during acute inflammation, atherosclerosis and metastasis (Cai et al., 2012; Dyer et al., 2014). A common hallmark of these diseases is vascular hyperpermeability, causing plasma proteins or cells to enter the surrounding tissue, leading to the formation of edema, plaques or metastases (Kumar et al., 2009; Reymond et al., 2013). However, the precise function of BMP signaling in the regulation of endothelial cell permeability remains elusive. We have investigated whether BMP signal transduction controls the permeability of human umbilical vein endothelial cell (HUVEC) monolayers and demonstrate that BMP6 induces hyperpermeability by promotion of VE-cadherin internalization as well as c-Src-mediated tyrosine phosphorylation. Furthermore, we identify VE-cadherin as a novel regulator of vascular BMP signal transduction and show that VE-cadherin physically associates with the BMP type I receptor activin receptor-like kinase 2 (ALK2, also known as ACVR1) and the BMP type II receptor (BMPRII, or BMPR2) in a BMP6-dependent manner and that it stabilizes receptor complex formation.

## RESULTS

### BMP6 induces hyperpermeability and disruption of AJ architecture

**Fig. 1.**
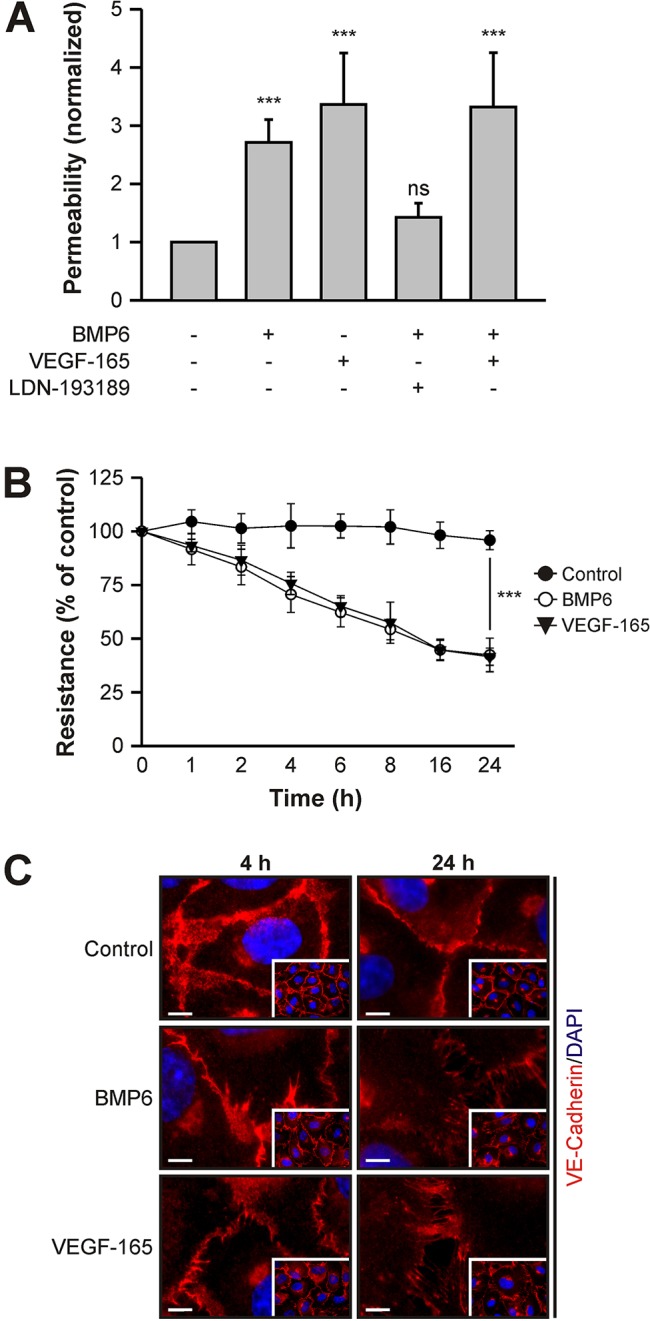
**BMP6 induces vascular permeability *in vitro*.** (A) BMP6 increases endothelial cell monolayer solute permeability. HUVECs seeded in transwell inserts were subjected to 10 nM BMP6 or 2 nM VEGF-165 and, as indicated, pharmacological inhibitor (0.5 µM LDN-193189) treatment for 24 h and *in vitro* transendothelial solute permeability was measured by FITC-Dextran flux. Mean±s.e.m. normalized to untreated control cells from ten biological replicates in three independent experiments. (B) BMP6 stimulation decreases transendothelial electrical resistance (TEER). HUVECs were seeded in transwell inserts and stimulated with growth factors for 24 h. At the indicated time points, TEER was measured. Mean±s.d. normalized to untreated control cells from nine biological replicates in three independent experiments. (A,B) ***P≤0.001; ns, not significant. (C) Immunocytochemical staining of VE-cadherin in HUVECs treated with growth factors for 4 and 24 h. Main images are representative regions from the insets. Scale bars: 5 µm.

To assess the effects of BMP signaling on vascular permeability, we measured solute flux across an endothelial cell monolayer. HUVECs were seeded onto collagen-coated transwell inserts and stimulated with BMP6 or VEGF-165 (a variant of VEGF-A) for 24 h. Subsequently, flux of high molecular weight FITC-Dextran across the monolayer was measured. We found that BMP6 stimulation led to a 2.7-fold increase in paracellular permeability, similar to the potent permeability-inducing growth factor VEGF-165 (Fig. 1A). When a pharmacological BMP type I receptor kinase inhibitor (LDN-193189) (Cuny et al., 2008) was administered 60 min before BMP6 stimulation, BMP6-mediated permeability was inhibited, suggesting that active BMPRI kinase is important for this process. Thus, we hypothesized that BMP6-induced permeability required signal transduction downstream of the BMP receptors. A co-stimulation with BMP6 and VEGF-165 showed no synergistic effects. These data suggested that BMP signaling specifically induced permeability of an endothelial cell monolayer.

We next addressed the kinetics of BMP-induced permeability by measuring changes in transendothelial electrical resistance (TEER). Electrical resistance decreased upon BMP6 treatment in a time- and dose-dependent manner (Fig. 1B, Fig. S1). A significant reduction (*P*<0.05) was detected as early as 2 h after stimulation and progressed over time, resulting in a 68±8% (mean±s.d.) reduction after 24 h compared with untreated control cells (Fig. 1B). Treatment of a HUVEC monolayer with VEGF-165 showed similar effects (68±4%). Thus, both methods of choice demonstrated a strong increase in permeability upon BMP6 stimulation.

Alterations of AJ architecture and, in particular, the localization of VE-cadherin are the main regulators of vascular permeability. Hence, we determined whether BMP6 treatment affects VE-cadherin localization. A confluent HUVEC monolayer was stimulated with BMP6 or VEGF-165 for 4 or 24 h and VE-cadherin was visualized by immunofluorescence staining. VE-cadherin staining in control cells showed close localization between neighboring cells, indicating a narrow AJ architecture (Fig. 1C). BMP6 stimulation led to ‘jagged’ VE-cadherin staining at 4 h and pronounced disruption of cell–cell junctions at 24 h. VEGF-165 treatment resulted in a comparable staining pattern. This demonstrated that BMP6 induces changes in AJ architecture reflected in a jagged, rather than narrow, borderline of adjacent cells. Taken together, our data showed that BMP6 treatment induces endothelial cell hyperpermeability and the disruption of AJs.

### BMP6 triggers internalization of VE-cadherin

**Fig. 2.**
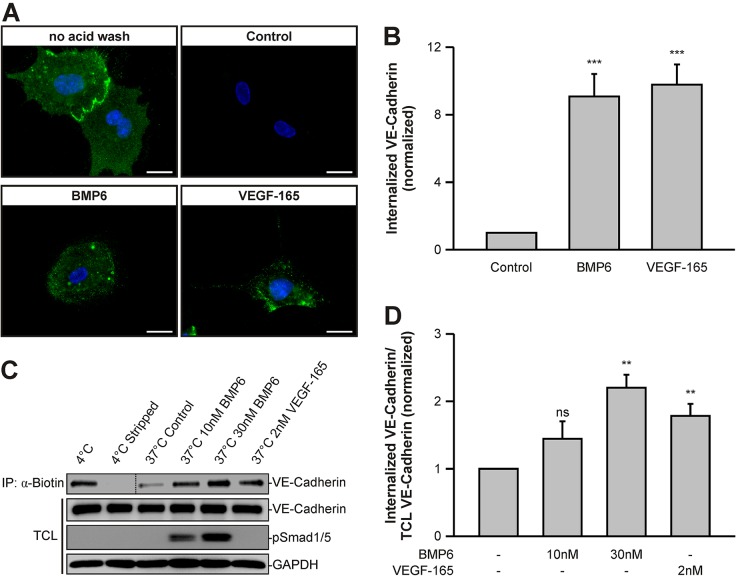
**BMP6 stimulation increases internalization of VE-cadherin.** (A) BMP6 increases the internalization rate of cell-surface-labeled VE-cadherin. HUVECs were incubated with the VE-cadherin extracellular domain-targeting antibody BV6 at 4°C. VE-cadherin internalization was monitored by uptake of BV6 antibody upon growth factor treatment for 60 min at 37°C. Remaining cell-surface antibodies, visible in ‘no acid wash’ conditions, were washed away with a mild acid solution and internalized VE-cadherin antibodies were visualized in fixed cells by addition of a fluorophore-coupled secondary antibody (green). Blue, DAPI. Scale bars: 20 µm. (B) Quantitation of VE-cadherin vesicles visible in A, normalized to untreated control cells. Mean±s.d. from three independent experiments. (C) BMP6 triggers internalization of cell-surface biotinylated VE-cadherin. HUVEC surface proteins were biotinylated at 4°C and growth factor-induced endocytosis was allowed to occur for 60 min at 37°C. Subsequently, remaining cell-surface biotin, which is visible in 4°C versus 4°C stripped samples, was stripped at 4°C and cells were rinsed and solubilized. Internalized biotinylated proteins were subjected to immunoprecipitation and samples were blotted as indicated. Total cell lysates (TCLs) represent samples before precipitation. Dotted line indicates merge of the same, but differentially exposed blot (left, 10 s; right, 60 s). (D) Quantitation of VE-cadherin signal intensities depicted in C and normalized to TCL VE-cadherin and untreated control cells. Mean±s.e.m. from four independent experiments. (B,D) **P≤0.005, ***P≤0.001; ns, not significant.

An increase in vascular permeability and concomitant disruptions of cell–cell contacts have been linked to internalization into subcellular compartments and subsequent recycling or degradation of VE-cadherin (Gavard and Gutkind, 2006). We therefore determined whether BMP stimulation modulates the rate of VE-cadherin endocytosis. We labeled VE-cadherin on sparse and confluent HUVEC cultures using a specific monoclonal antibody (BV6) targeting an extracellular epitope of VE-cadherin (Corada et al., 2001). The cells showed a cell-surface staining that was sensitive to acid wash (Fig. 2A, Fig. S2A). Although the BV6 antibody has been described as increasing endothelial cell permeability (Corada et al., 2001), we did not detect significant amounts of intracellular VE-cadherin vesicles in control cells (Fig. 2A). This suggests that the antibody exerts its effects by inhibition of VE-cadherin clustering at 37°C. However, upon 60 min of BMP6 treatment, an acid-resistant intracellular VE-cadherin staining was observed in sparse HUVECs, comparable to that observed under VEGF-165 stimulation conditions. Quantitation of labeled VE-cadherin uptake normalized to untreated control cells revealed similar effects for BMP6 and VEGF-165 treatment (Fig. 2B). Upon 4 h of BMP6 or VEGF-165 treatment, similar observations were made in confluent HUVECs (Fig. S2). From this we conclude that BMP6 induces VE-cadherin internalization.

To further investigate BMP6-induced internalization of VE-cadherin, we biotinylated cell-surface proteins using Sulfo-NHS-SS biotin (Gabriel et al., 2009). Subsequently, we allowed endocytosis to occur for 60 min, stripped the remaining extracellular biotin and measured the levels of biotinylated, internalized VE-cadherin. Removal of biotinylated, cell-surface VE-cadherin was very efficient, as demonstrated by stripping controls (stripping efficiency of 92±7%, mean±s.d.) (Fig. 2C). Control cells exhibited relatively low levels of internalized VE-cadherin (18% of total VE-cadherin) and levels were increased upon BMP6 stimulation (50% of total) in a dose-dependent manner. Quantitations showed that addition of 10 nM BMP6 resulted in a 1.4-fold increase in biotinylated internalized VE-cadherin compared with untreated control cells, while 30 nM BMP6 led to a 2.2-fold increase (Fig. 2D). Again, similar effects were observed upon 2 nM VEGF-165 treatment (1.8-fold). These data showed that addition of BMP6 to endothelial cells results in increased VE-cadherin internalization.

### BMP6 induces phosphorylation of VE-cadherin at Tyr685 by activating c-Src

**Fig. 3.**
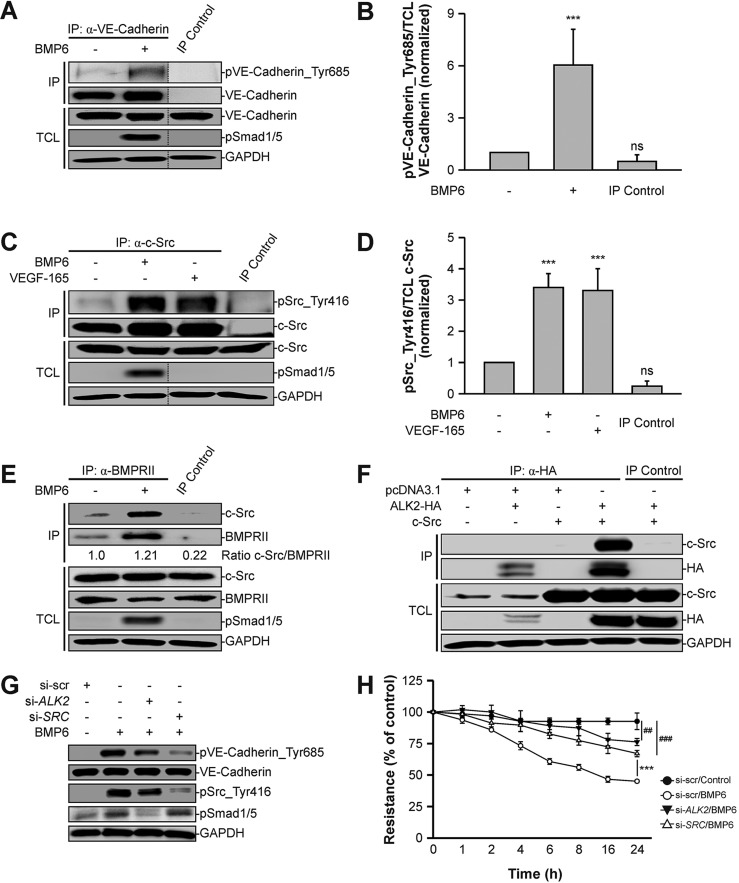
**BMP6 induces phosphorylation of VE-cadherin via activation of c-Src.** (A) VE-cadherin is phosphorylated at Tyr685 upon BMP6 treatment. VE-cadherin was immunoprecipitated with a VE-cadherin-specific antibody from confluent HUVECs treated with BMP6 for 30 min and samples were blotted as indicated. Normal IgG antibody served as the immunoprecipitation (IP) control. TCLs represent lysates not subjected to IP. Dotted lines indicate where samples from the same blot have been omitted. (B) Quantitation of pVE-cadherin_Tyr685 signal intensities depicted in A and normalized to TCL VE-cadherin and untreated control cells. Mean±s.d. from four independent experiments. (C) c-Src kinase is activated upon BMP6 treatment. c-Src was immunoprecipitated with a c-Src-specific antibody from confluent HUVECs treated with growth factors for 15 min. Normal IgG antibody served as an IP control. (D) Quantitation of pSrc_Tyr416 signal intensities shown in C and normalized to TCL c-Src and untreated control cells. Mean±s.d. from three independent experiments. (E) BMPRII interacts endogenously with c-Src. BMPRII was immunoprecipitated with a BMPRII-specific antibody from confluent HUVECs stimulated with BMP6 for 30 min and samples were blotted as indicated. Quantitation shows the c-Src versus BMPRII IP signal intensity ratio. Normal IgG antibody served as an IP control. (F) ALK2 associates with c-Src. HEK293T cells were transfected with ALK2-HA and c-Src, and HA-tagged ALK2 was immunoprecipitated with an HA-specific antibody. Normal IgG antibody served as IP control. (G) ALK2 and c-Src are required for BMP-induced phosphorylation of VE-cadherin. HUVECs were transfected with siRNA targeting either nonspecific sequences (si-scr), human ALK2 (si-*ALK2*) or human c-Src (si-*SRC*) and treated with BMP6 for 30 min. Cells were lysed and blotted as indicated. (H) BMP6-induced permeability is mediated by ALK2 and c-Src. HUVECs were transfected with si-scr, si-*ALK2* or si-*SRC*, seeded in transwell inserts and stimulated with BMP6 for 24 h. At the indicated time points, TEER was measured. Mean±s.d. normalized to untreated control cells from three independent experiments. ***P≤0.001; ns, not significant. For BMP6-treated si-*ALK2* and si-*SRC* versus untreated si-scr: ^##^*P*≤0.005, ^###^*P*≤0.001.

Besides its internalization, post-translational modifications of VE-cadherin have been shown to regulate vascular permeability. Recently, it was demonstrated that phosphorylation of VE-cadherin at Tyr685 controls the opening of endothelial cell junctions *in vivo* (Wessel et al., 2014). Hence, we analyzed whether BMP signal transduction alters the phosphorylation status of VE-cadherin. We immunoprecipitated VE-cadherin from BMP6-stimulated or untreated HUVECs and analyzed the levels of VE-cadherin phosphorylation at Tyr685 using a phospho-specific antibody. Upon 30 min of BMP6 treatment, we observed a 6-fold increase in the levels of phosphorylated VE-cadherin (Fig. 3A,B). Thus, we concluded that BMP6 induces the phosphorylation of VE-cadherin at Tyr685.

Next, we investigated the molecular mechanism underlying this phosphorylation event. It has been shown that c-Src specifically phosphorylates VE-cadherin at Tyr685 upon VEGF-165 stimulation *in vitro* (Wallez et al., 2007). We tested whether BMP6 is able to activate c-Src in endothelial cells. We immunoprecipitated c-Src from BMP6-stimulated or untreated HUVECs and determined the levels of phosphorylation of c-Src at Tyr416, an indicator for c-Src kinase activation. Control cells exhibited a low baseline phosphorylation, whereas 15 min of BMP6 treatment resulted in a significant increase in the levels of phosphorylated c-Src (*P*≤0.001; Fig. 3C). VEGF-165 was used as a positive control as it has been demonstrated to activate c-Src in cultured endothelial cells (Ha et al., 2008). Quantitation revealed a 3.4-fold and a 3.3-fold increase in phosphorylated c-Src levels compared with control HUVECs for BMP6 and VEGF-165, respectively (Fig. 3D). This showed that BMP signal transduction triggers the activation of c-Src. Moreover, we demonstrated that endogenous BMPRII and c-Src co-immunoprecipitate in HUVECs and upon overexpression in HEK293T cells (Fig. 3E, Fig. S3A). A similar approach demonstrated an association between ALK2, which is the high-affinity BMPRI for BMP6, and c-Src upon overexpression in HEK293T cells (Fig. 3F). These findings suggested that c-Src interacts with the BMP–receptor complex and its activity is regulated by BMP signal transduction.

To further dismantle the interplay of BMP signaling, we performed knockdown experiments using siRNAs targeting *ALK2* or *SRC* transcripts (Fig. S3B). We demonstrated that upon BMP6 treatment, VE-cadherin was phosphorylated at Tyr685 and this phosphorylation was partially abrogated by knockdown of *ALK2* (53% reduction) or *SRC* (85% reduction) (Fig. 3G). Similar, but less pronounced, reductions were obtained with respect to c-Src phosphorylation at Tyr416 (*ALK2* knockdown, 30% reduction; *SRC* knockdown, 67% reduction). Phosphorylation of SMAD1/5 was abrogated by knockdown of *ALK2*, but was unaffected in *SRC* knockdown conditions. Furthermore, we observed comparable results using small-molecule inhibitors targeting either ALK2 kinase (K02288) (Sanvitale et al., 2013) or Src family kinases (PP2) (Hanke et al., 1996) (Fig. S3C). Taken together, these data demonstrated that BMP signaling induces phosphorylation of VE-cadherin at Tyr685 via BMP receptor-mediated activation of c-Src.

Since VE-cadherin phosphorylation is associated with increased vascular permeability, we analyzed BMP6-induced changes in TEER upon siRNA-mediated knockdown of *ALK2* or *SRC*. BMP6 treatment triggered a decrease in electrical resistance in control cells in a time-dependent manner (Fig. 3H). However, upon knockdown of *ALK2* or *SRC* this effect was significantly reduced, yet partial permeabilization still occurred. Similar results were obtained when ALK2 kinase was inhibited by K02288 or c-Src kinase activity was blocked by PP2 (Fig. S3D). Taken together, our findings suggest that BMP6-induced permeability requires ALK2 and, at least partly, c-Src.

### VE-cadherin facilitates BMP signal transduction

**Fig. 4.**
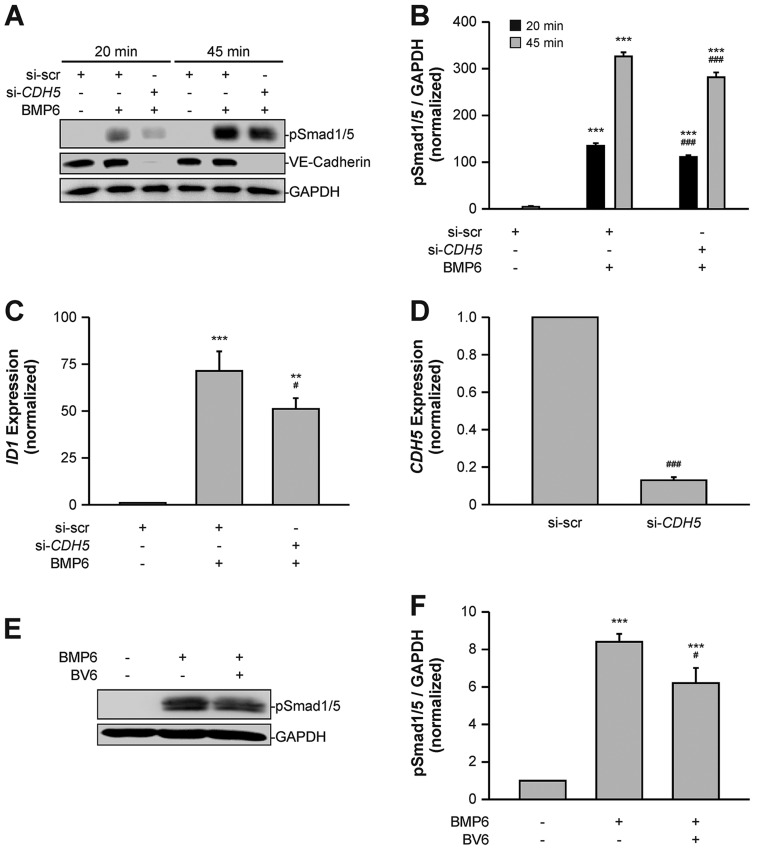
**VE-cadherin regulates endothelial BMP signaling.** (A) VE-cadherin is required for efficient BMP signal transduction. HUVECs were transfected with si-scr or si-*CDH5* and treated with BMP6 for the indicated times. Cells were lysed and blotted as indicated. (B) Quantitation of pSMAD1/5 signal intensities shown in A and normalized to GAPDH and untreated control cells. Mean±s.d. from three independent experiments. (C) VE-cadherin is required for optimal activation of SMAD1/5 target gene expression. HUVECs were transfected with si-scr or si-*CDH5* and treated with BMP6 for 60 min. *ID1* mRNA expression was determined using qRT-PCR and normalized to *GAPDH* control mRNA expression and to untreated control cells. Mean±s.e.m. from three independent experiments. (D) *CDH5* mRNA expression was determined after siRNA transfection using qRT-PCR and normalized to *GAPDH* control mRNA expression and to si-scr-treated control cells. Mean±s.e.m. from three independent experiments. (E) VE-cadherin clusters are needed for efficient BMP signaling. HUVECs were incubated with the VE-cadherin extracellular domain-targeting antibody BV6 or isotype control antibody and treated with BMP6 for 45 min. Cells were lysed and blotted as indicated. (F) Quantitation of pSMAD1/5 signal intensities depicted in E and normalized to GAPDH and untreated control cells. Mean±s.d. from three independent experiments. **P≤0.005, ***P≤0.001. For BMP6-treated si-scr and si-*CDH5*: ^#^*P*≤0.05, ^###^*P*≤0.001.

As we demonstrated an effect of BMP signal transduction on VE-cadherin internalization and modification, we next examined whether VE-cadherin in turn modulates BMP signaling in endothelial cells. To assess the impact of VE-cadherin on BMP signaling, we performed knockdown experiments in HUVECs using siRNA targeting VE-cadherin (*CDH5*) mRNA (si-*CDH5*). The knockdown efficiency at the protein level, as detected by western blot, was 95±2% (mean±s.d.) compared with cells transfected with non-targeting siRNA (si-scr) (Fig. 4A). Upon stimulation with BMP6, a time-independent decrease in the levels of phosphorylated (p)SMAD1/5 was detected (Fig. 4A). Quantitation of pSMAD1/5 levels revealed significant 18% (*P*≤0.001) and 14% (*P*≤0.001) reductions between si-scr-treated and si-*CDH5*-treated cells upon 20 or 45 min treatment, respectively (Fig. 4B). This suggested that VE-cadherin acts as a positive regulator of endothelial BMP signal transduction.

As levels of pSMAD1/5 were decreased in VE-cadherin knockdown conditions, we analyzed whether SMAD1/5-dependent *ID1* expression was also altered (Korchynskyi and ten Dijke, 2002). We treated HUVECs with si-scr or si-*CDH5*, stimulated with BMP6 for 60 min and then determined *ID1* mRNA expression levels by qRT-PCR. We observed an increase in *ID1* expression levels between BMP6-treated and unstimulated control cells, which was significantly decreased by 28% (*P=*0.042) upon treatment with si-*CDH5* (Fig. 4C). Treatment of HUVECs with si-*CDH5* resulted in an 87±2% (mean±s.d.) reduction of *CDH5* mRNA (Fig. 4D). These data showed that the transcriptional activity of SMAD1/5 is reduced upon knockdown of VE-cadherin.

The major function of VE-cadherin is to form clusters in a transhomophilic manner between neighboring cells and to facilitate the formation of cell–cell junctions. Hence, we examined whether proper clustering of VE-cadherin at AJs is needed for endothelial BMP6 signaling. The BV6 antibody has been described to block homophilic interactions and dismantle VE-cadherin clusters (Corada et al., 2001). Confluent HUVEC cultures treated with BMP6 in the presence of the VE-cadherin-blocking antibody BV6 demonstrated decreased pSMAD1/5 levels compared with cells treated with a nonspecific isotype IgG antibody (Fig. 4E). Quantitation of pSMAD1/5 revealed a significant reduction by 26% (*P*≤0.001) upon BV6 treatment (Fig. 4F). Taken together, these findings demonstrated that VE-cadherin positively regulates BMP signal transduction and that proper VE-cadherin clusters at cell–cell junctions are required for efficient signaling.

### VE-cadherin associates with the BMP–receptor complex

**Fig. 5.**
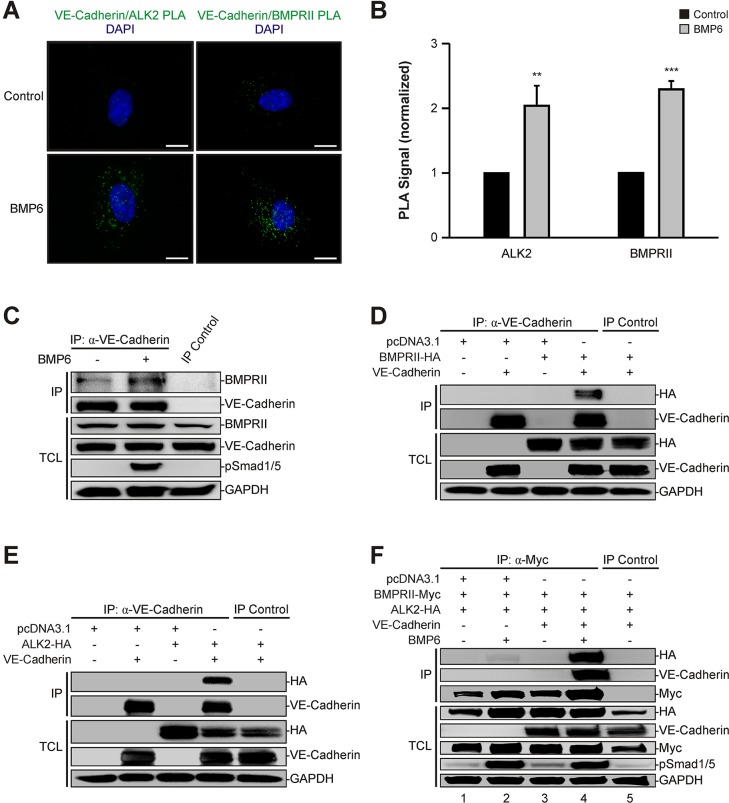
**VE-cadherin interacts with BMP receptors.** (A) *In situ* proximity ligation assay (PLA) of VE-cadherin and the BMP receptors ALK2 and BMPRII. HUVECs were treated with BMP6 for 60 min and association of VE-cadherin and ALK2 or BMPRII was visualized using an *in situ* PLA (green). DAPI, blue. Scale bars: 10 µm. (B) Quantitation of VE-cadherin–ALK2 and VE-cadherin–BMPRII heteromers shown in A. Mean±s.d. from three independent experiments. (C) VE-cadherin interacts endogenously with BMPRII. VE-cadherin was immunoprecipitated with a VE-cadherin-specific antibody from confluent HUVECs stimulated with BMP6 for 60 min and samples were blotted as indicated. Recombinant protein A-Sepharose beads served as an IP control. TCLs represent lysates not subjected to immunoprecipitation. (D) VE-cadherin associates with BMPRII upon overexpression. HEK293T cells were transfected with VE-cadherin and BMPRII-HA, and VE-cadherin was immunoprecipitated with a VE-cadherin-specific antibody. Normal IgG antibody served as IP control. (E) VE-cadherin associates with ALK2 upon overexpression. HEK293T cells were transfected with VE-cadherin and ALK2-HA, and VE-cadherin was immunoprecipitated with a VE-cadherin-specific antibody. Normal IgG antibody served as IP control. (F) VE-cadherin facilitates BMP receptor complex formation. HEK293T cells were transfected with BMPRII-Myc and ALK2-HA in the absence or presence of co-expressed VE-cadherin. Cells were stimulated with BMP6 for 45 min, followed by immunoprecipitation of Myc-tagged BMPRII with a Myc-specific antibody. Mouse IgG isotype antibody served as IP control. **P≤0.005, ***P≤0.001.

We have shown that VE-cadherin regulates BMP6-induced phosphorylation of SMAD1/5 and downstream transcriptional activity. It has previously been reported that VE-cadherin forms complexes with structurally related TGF-β receptors (Rudini et al., 2008) and thus we hypothesized that VE-cadherin might also interact with the related BMP receptors. To test this in intact cells, we made use of an *in situ* proximity ligation assay (PLA). HUVECs were either stimulated with BMP6 for 60 min or left untreated. Subsequently, cells were fixed and probed with antibodies targeting intracellular epitopes of either ALK2 or BMPRII in combination with VE-cadherin. Heteromers were visualized by proximity-dependent ligation of species-specific, oligonucleotide-conjugated secondary antibodies, followed by incubation with a fluorescent probe. Cells that were not treated with ligand had few ALK2–VE-cadherin or BMPRII–VE-cadherin heteromers (Fig. 5A), whereas cells exposed to BMP6 showed a higher number of heteromers for both ALK2–VE-cadherin (2-fold, *P=*0.004) and BMPRII–VE-cadherin (2.3-fold, *P*≤0.001) heteromers (Fig. 5B). This suggested that VE-cadherin physically associates with BMP type I and type II receptors in a ligand-dependent manner.

To confirm this interaction, we immunoprecipitated VE-cadherin from confluent HUVECs that were stimulated with BMP6 for 60 min or left untreated and then were analyzed for associated BMPRII. Complex formation between VE-cadherin and BMPRII was already detectable in control HUVECs, but the interaction increased upon ligand addition (Fig. 5C). These VE-cadherin–BMPR complexes were further validated by overexpression of HA-tagged BMPRII or ALK2 in combination with VE-cadherin in HEK293T cells followed by immunoprecipitation of VE-cadherin (Fig. 5D,E).

BMP signal transduction is initiated upon ligand-induced activation of the heteromeric BMP type I and type II receptor complex. As VE-cadherin interacted with both receptor types in a ligand-dependent fashion and positively regulated downstream signaling, we hypothesized that VE-cadherin is needed for proper BMP receptor complex assembly. To address this question, we overexpressed HA-tagged ALK2 and Myc-tagged BMPRII in the absence or presence of co-expressed VE-cadherin in HEK293T cells and stimulated with BMP6 for 45 min. Subsequently, BMPRII-Myc was immunoprecipitated and protein levels of associated ALK2-HA and VE-cadherin were determined. In the absence of VE-cadherin, a low degree of receptor oligomerization was observed in BMP6-treated cells (Fig. 5F, lanes 1 and 2). This interaction was increased remarkably when VE-cadherin was co-expressed and ligand-dependent complex formation between ALK2, BMPRII and VE-cadherin was detected (Fig. 5F, lane 4). These data demonstrated that VE-cadherin physically associates with ALK2 and BMPRII in a ligand-dependent manner in endothelial cells and stabilized BMP receptor complex formation.

## DISCUSSION

Vascular BMP signaling regulates physiological as well as pathological processes in the endothelium, yet a detailed molecular analysis of its influence on vascular permeability is missing. Here, we provide evidence that BMP6 increases permeability *in vitro* and unravel a hitherto undescribed regulatory mechanism of vascular BMP signal transduction involving VE-cadherin.

We measured solute flux and electrical resistance across a HUVEC monolayer to assess alterations in permeability and showed that BMP6 induces hyperpermeability similar to VEGF in a time- and dose-dependent manner. Pharmacological inhibition of the BMP6 type I receptor ALK2 demonstrated that this process requires activation of the BMP receptor complex. These findings are in line with a recent study showing that BMP2 induces permeability of retinal endothelial cells (Hussein et al., 2014). BMP2 was upregulated in human and murine diabetic retinas and promoted permeability in a time-dependent manner. Moreover, BMP2-induced hyperpermeability was also reported in lung epithelial cells (Helbing et al., 2013). BMP2 was upregulated in injured lung epithelium and caused increased solute permeability of cultured bronchial epithelial cells via activation of SMAD1/5-ID1 signaling and downregulation of E-Cadherin. The authors of that study concluded that excessive BMP activity is involved in the disruption of epithelial barrier function after lung injury. This assumption is consistent with other published studies that relate BMP signaling to disturbed barrier function. During acute inflammation, increased vascular permeability is one of the first events to take place in order for plasma proteins, fluids and immune cells to reach the injured tissue. Several reports indicate that BMP signaling is activated during inflammation and atherosclerosis and increased monocyte adhesion to endothelial cells has been observed upon BMP treatment (Rosendahl et al., 2002; Sorescu et al., 2003, 2004; Helbing et al., 2011; Derwall et al., 2012; Lee et al., 2012).

Another process that involves opening of the endothelial barrier is metastasis. During metastasis, disseminated cancer cells extravasate from the bloodstream to invade surrounding tissues and organs. It is well known that BMPs enhance the invasiveness of cancer cells *in vitro* and *in vivo* (Thawani et al., 2010) and BMP6 expression correlates with the metastatic potential of prostate cancer (Hamdy et al., 1997). Furthermore, patients with hereditary pulmonary arterial hypertension (PAH) exhibit endothelial cell dysfunction and vascular leakage that have been linked to heterozygous *BMPR2* mutations (Prewitt et al., 2015). These observations highlight the implications of perturbed BMP signaling in diseases associated with impaired endothelial barrier function. However, *in vivo* studies demonstrating the importance of BMP-induced vascular permeability are still lacking, and future studies are required to gain further mechanistic insight.

In our study, we demonstrated that endothelial AJ architecture is dramatically altered upon BMP6 or VEGF stimulation, as VE-cadherin staining revealed a jagged borderline with visible openings between neighboring cells. These types of cell–cell contacts have been termed remodeling junctions (Huveneers et al., 2012), and changes in VE-cadherin localization at AJs have been repeatedly attributed to the control of vascular permeability (Dejana et al., 2008). A well-described cause for these changes is the internalization of VE-cadherin into intracellular vesicles, thereby removing it from the cell surface. This results in the reversible disruption of cell–cell junctions until surface levels of VE-cadherin are restored. We show that BMP6 triggers the internalization of VE-cadherin in a dose-dependent manner in HUVECs at similar rates as VEGF. Gavard and Gutkind (2006) reported that VEGF induces β-arrestin 2-dependent endocytosis of VE-cadherin and showed that a specific serine residue (Ser665) within the cytoplasmic domain of VE-cadherin was phosphorylated upon VEGF treatment, served as a docking site for β-arrestin 2 and regulated solute permeability in cultured endothelial cells. Although β-arrestin 2-mediated endocytosis has been mainly linked to G-protein-coupled receptor (GPCR) signaling, a few studies describe its function in the internalization of other cell-surface proteins, such as insulin-like growth factor I receptor (IGF1R) (Lin et al., 1998). Interestingly, a study by Chen et al. (2003) provided evidence for β-arrestin 2-mediated endocytosis of the TGF-β type III receptor betaglycan (also known as TGFβR3). They reported that internalization was initiated upon phosphorylation of a specific threonine residue (Thr841) within the cytoplasmic domain of betaglycan by the TGF-β type II receptor kinase. This finding suggests that serine/threonine kinase receptors of the TGF-β family promote β-arrestin 2-dependent endocytosis by phosphorylation of specific residues within their targets. Whether VE-cadherin contains serine/threonine residues that can be phosphorylated by the BMP receptors and whether these modifications initiate β-arrestin-mediated internalization to control vascular permeability, similar to VEGF, remains unknown at present.

Another mechanism that impairs endothelial barrier function is the tyrosine phosphorylation of VE-cadherin, which promotes the remodeling of AJs. Multiple permeability-increasing agents, such as VEGF (Esser et al., 1998), TNF-α (Angelini et al., 2006) or histamine (Andriopoulou et al., 1999), induce tyrosine phosphorylation of VE-cadherin *in vitro*. However, conflicting data exist about which specific VE-cadherin tyrosine residue (Tyr658, Tyr685, Tyr731) becomes phosphorylated in cultured endothelial cells (Potter et al., 2005; Wallez et al., 2007; Adam et al., 2010). Using knock-in mouse models, two recent studies elegantly revealed that VE-cadherin phosphorylation at Tyr685 controls vascular permeability, whereas phosphorylation at Tyr731 regulates leukocyte extravasation (Sidibe et al., 2014; Wessel et al., 2014). Thus, we addressed whether BMP signaling induces phosphorylation of VE-cadherin and showed that, upon BMP6 stimulation of HUVECs, VE-cadherin is phosphorylated at Tyr685. A peptide-mapping approach on the cytoplasmic domain of VE-cadherin indicated that Tyr685 is specifically phosphorylated by c-Src (Wallez et al., 2007). This finding is in line with several other studies that demonstrate an involvement of c-Src in VEGF-induced vascular permeability (Eliceiri et al., 1999; Gavard et al., 2008; Ha et al., 2008).

Here, we provide evidence for a BMP6-induced activation of c-Src in an ALK2 kinase-dependent manner in HUVECs. We show that ALK2 and c-Src are required for phosphorylation of VE-cadherin at Tyr685. Moreover, we demonstrate a physical interaction of c-Src and ALK2 as well as BMPRII and that c-Src becomes activated upon receptor complex formation and in turn phosphorylates downstream targets at specific tyrosine residues. These observations are in contrast to a recent study demonstrating that mice carrying heterozygous null mutations of *Bmpr2* in pulmonary endothelial cells show constitutive activation of Src kinase and that this effect can be reversed by BMP2 stimulation (Prewitt et al., 2015). Furthermore, BMP signaling was shown to inhibit Src activation in pulmonary smooth muscle cells, but large differences in inhibition potential were seen between different BMPs, and BMP7, a member of the same BMP subgroup as BMP6, only had a mild effect on Src kinase inhibition (Wong et al., 2005). The authors also showed a BMPRII–Src interaction using a yeast two-hybrid approach and overexpression in HEK293T cells, yet this interaction has thus far not been reproduced in any other cell type. Hence, these studies and our findings strongly indicate that BMP-dependent regulation of Src activity is ligand- and context-dependent and might require hitherto unidentified effectors.

However, in line with the study by Prewitt et al. (2015), we assume that Src activity is, at least in part, regulated by BMPRII. As we and others have shown previously, BMP and TGF-β type II receptors carry dual kinase activity (Lawler et al., 1997), leading to cis and trans phosphorylation at serine, threonine and tyrosine residues. Tyrosine-phosphorylated BMPRII might act as a docking site for c-Src, as previously suggested by Wong et al. (2005). Furthermore, *in silico* analysis shows that the C-terminal domain of BMPRII contains putative binding sites for Src homology 3 (SH3) domains that could explain the binding of c-Src to BMPRII in a tyrosine phosphorylation-independent manner. Future studies should address the exact nature of this interaction, especially considering the conflicting data on the resulting effects.

Besides describing its influence on endothelial cell permeability, we report a novel regulatory mechanism of vascular BMP signal transduction by VE-cadherin. We showed that VE-cadherin knockdown in HUVECs decreases BMP signaling activity and that proper VE-cadherin clustering at endothelial cell–cell junctions is required for efficient local BMP signal transduction. As VE-cadherin interacts with ALK2 and BMPRII in a ligand-dependent manner, we propose a stabilizing function of VE-cadherin for BMP receptor complexes in endothelial cell junctions. Interestingly, results from PLAs suggested that BMP receptors associate with VE-cadherin in intracellular vesicles. We have previously shown that both BMPRI and BMPRII undergo clathrin-mediated endocytosis, but caveolin-dependent internalization was only observed for BMPRII (Hartung et al., 2006). It has been reported that VE-cadherin is internalized via clathrin-coated pits by a p120-catenin-dependent mechanism (Xiao et al., 2005). Besides, VE-cadherin recruits caveolin-1 to endothelial cell junctions and phosphorylated caveolin-1 weakens VE-cadherin–catenin interactions and thus regulates endothelial barrier function (Kronstein et al., 2012). Furthermore, a recent study demonstrated that heterozygous null *Bmpr2* mutations affect Src-dependent, caveolin-dependent internalization as well as the phosphorylation of caveolin-1 (Prewitt et al., 2015). These observations indicate that BMP-induced permeability may be differentially regulated by clathrin- and caveolin-dependent endocytosis mechanisms.

In this study, we show that VE-cadherin acts as a positive regulator of endothelial BMP signal transduction, which is consistent with a previous study describing a similar role of VE-cadherin in TGF-β signaling (Rudini et al., 2008). It was shown that VE-cadherin interacts with the TGF-β receptor complex and facilitates oligomerization of the complex in a ligand-dependent fashion (Rudini et al., 2008). Moreover, using VE-cadherin knockout endothelial cells, the authors showed that VE-cadherin acts as a positive regulator of endothelial TGF-β signal transduction and found that proper VE-cadherin clustering is needed for efficient signaling. These observations suggest that VE-cadherin physically associates with receptors of the TGF-β family to stabilize their oligomerization and increase downstream signaling. This stands in contrast to the VE-cadherin-mediated inhibition of VEGF-VEGFR2 signal transduction in confluent endothelial cells. It has been shown that VEGFR2 is dephosphorylated and its internalization strongly reduced upon association with VE-cadherin (Lampugnani et al., 2006). The net effect was an inhibition of cell proliferation by decreasing efficient VEGFR2 signaling. The authors proposed that this regulatory mechanism modulated the capacity of cells to proliferate and survive in a density-dependent manner, thereby controlling contact inhibition of growth *in vitro*. In combination with the finding that TGF-β-induced inhibition of cell migration and proliferation was enhanced in the presence of VE-cadherin (Rudini et al., 2008), these observations strongly support the idea that VE-cadherin modulates growth factor receptor signaling in favor of vessel stability.

However, the BMP6-induced hyperpermeability and the positive effects of VE-cadherin on BMP signaling *in vitro* that we describe seem to contradict this idea. The effects of BMP6 on endothelial cells are less well characterized compared with other BMP family members, such as BMP2 and BMP9. Dose-dependent binding of distinct BMP ligands causes the recruitment and a unique mode of oligomerization of specific BMP receptors, which is crucial for the induction of individual downstream signaling pathways and a hallmark of this signal transduction pathway (Ehrlich et al., 2012). It has been reported that BMP6 activates endothelial cells by induction of cell migration and tube formation (Valdimarsdottir et al., 2002). These findings are in line with our results showing BMP6-induced activation with respect to HUVEC permeability. However, BMP9 has been reported to inhibit the proliferation and migration of endothelial cells induced by basic fibroblast growth factor (bFGF, also known as FGF2) (Scharpfenecker et al., 2007), indicating ligand-dependent outcomes. Similar phenomena were observed upon TGF-β stimulation in several studies. It has been shown that upon TGF-β inhibition by overexpression of soluble endoglin, mouse retinal permeability was increased (Walshe et al., 2009). However, Behzadian et al. (2001) demonstrated that TGF-β stimulation of bovine retinal cells induces hyperpermeability via activation of matrix metalloproteinase 9 (MMP9) (Behzadian et al., 2001). Moreover, TGFβ1 has been shown to regulate bFGF- and VEGF-induced endothelial cell invasion and tube formation in a dose-dependent manner. High doses had an inhibitory effect, whereas low doses potentiated the effects of bFGF and VEGF in bovine microvascular cells (Pepper et al., 1993). Interestingly, Goumans et al. (2002) provided evidence that the activation state of endothelial cells is regulated by TGF-β *in vitro.* Cell migration and proliferation were activated by ALK1–Smad1/5 signaling, whereas ALK5–Smad2 had an inhibitory effect. These observations demonstrate that vascular BMP/TGF-β signal transduction is ligand-, dose- and context-dependent, and that further clarification is needed, especially considering the regulation by VE-cadherin.

**Fig. 6.**
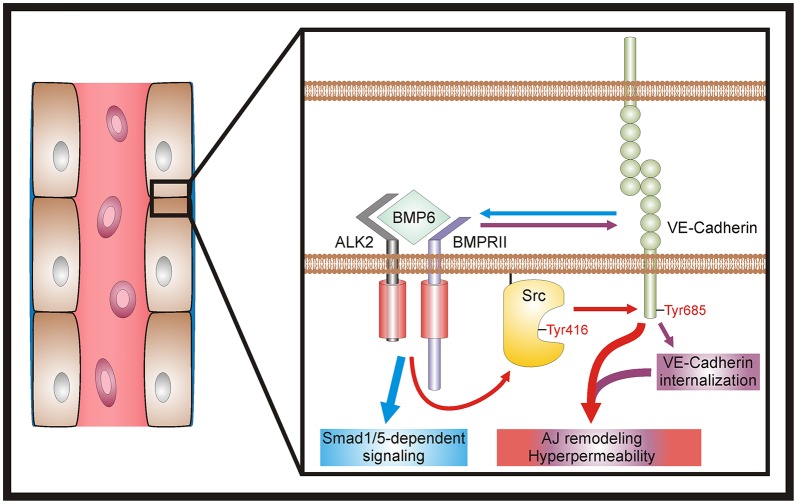
**Model of BMP6-induced vascular permeability.** At endothelial cell AJs, BMP6 induces receptor complex assembly, activates c-Src via phosphorylation at Tyr416 and promotes c-Src-dependent phosphorylation of VE-cadherin at Tyr685 (red arrows). In addition, BMP signaling induces VE-cadherin internalization (purple arrows). Collectively, these mechanisms trigger AJ remodeling and ultimately result in hyperpermeability (red and purple box). Moreover, VE-cadherin functions as a positive regulator of vascular BMP signal transduction. Clustered VE-cadherin is needed for efficient signaling and stabilizes the ALK2–BMPRII receptor complex (blue arrow), thus facilitating SMAD1/5-dependent signaling (blue box).

In the present study, we demonstrate that BMP6 induces endothelial cell permeability *in vitro* and provide mechanistic insight by showing that BMP signaling remodels AJ architecture by promoting the internalization and modification of VE-cadherin. Our study suggests that endothelial BMP signaling is modulated by VE-cadherin and controls barrier function (Fig. 6). Further studies should pave the way for novel therapies in the context of acute inflammation, atherosclerosis, metastasis and multiple other pathologies associated with increased vascular permeability.

## MATERIALS AND METHODS

### Reagents and antibodies

All chemicals were purchased from Sigma-Aldrich, unless otherwise indicated. Production of recombinant human BMP6 was described previously (Simic et al., 2006; Vukicevic et al., 2014). BMP6 used in the experiments has been produced under collaborative FP7 health project Osteogrow (grant agreement number 279239) by GMP manufacturing for clinical trials in patients for bone regeneration. The BMP6 producer cell line is CHO DG44, suspension adapted, transduced by a lentivirus vector with advanced safety features. Lentivirus vector was chosen for its unique features allowing highly efficient, multicopy, stable transduction of genes into growing and stationary cells (Lentigen Corporation). BMP6 was purified using heparin Sepharose, then phenyl Sepharose, and finally subjected to C18 RP-HPLC, analyzed under GMP conditions (Biochem) and found free of endotoxins and host cell proteins (SGS Vitrology). Investigational medicinal product dossier (IMPD) has been approved by national regulatory agencies in Austria and Croatia. In a standardized *in vitro* assay using C2C12 cells, BMP6 has been compared with protein from R&D Systems and had a similar biological potency. BMP6 was used at 10 nM, unless otherwise indicated. Recombinant human VEGF-165 was purchased from ImmunoTools and used at 2 nM. LDN-193139 was a kind gift of Paul B. Yu (Boston, USA) (Cuny et al., 2008) and Alex N. Bullock (Oxford, UK) kindly provided K02288 (Sanvitale et al., 2013).

The following antibodies were purchased from Cell Signaling Technology: rabbit anti-VE-cadherin (D87F2), rabbit anti-phospho-SMAD1/5 (Ser463/465) (41D10), rabbit anti-GAPDH (14C10), rabbit anti-phospho-Src family (Tyr416) (D49G4), rabbit anti-Src (36D10), rabbit anti-BMPR2, rabbit anti-ACVR1 (ALK2), mouse anti-Myc (9B11), mouse IgG1 isotype control (G3A1) and normal rabbit IgG. In addition, mouse anti-human VE-cadherin (BV6, Enzo Life Sciences), goat anti-VE-cadherin (C-19, Santa Cruz), rabbit anti-phospho-VE-cadherin (Tyr685, ECM Biosciences) and mouse anti-HA (HA-7, Sigma-Aldrich) were used. For western blot analysis, all antibodies were used at 1:1000.

### Cell culture and transfections

HUVECs were a kind gift from M. Lorenz, V. Stangl and A. Pries (Berlin, Germany) and cultured on gelatin-coated tissue culture ware (Greiner Bio-One) in M199 medium (Sigma-Aldrich; with Earle's salts and NaHCO_3_) supplemented with 20% fetal calf serum (FCS) (Biochrom), 50 µg/ml endothelial cell growth supplement (Corning), 25 µg/ml heparin, 2 mM L-glutamine, 100 U/ml penicillin and 0.1 mg/ml streptomycin (herein referred to as HUVEC growth medium) at 37°C and 5% CO_2_. HUVECs were used between passage two to five from isolation. Unless indicated otherwise, HUVECs were starved for 6 h prior to stimulation in M199 medium (with Earle's salts and NaHCO_3_) supplemented with 2 mM L-glutamine and 100 U/ml penicillin and 0.1 mg/ml streptomycin (herein referred to as HUVEC starvation medium).

Pharmacological inhibitors (LDN-193189, K02288 or PP2) were added 60 min prior to stimulation. Equal volumes of dimethylsulfoxide (DMSO) were used to treat control cells.

For knockdown, 40 nM commercially available siRNA against ALK2 (herein referred to as si-*ALK2*), c-Src (si-*SRC*) and VE-cadherin (si-*CDH5*) (ON-TARGETplus SMARTpool, GE Healthcare) was transfected in HUVECs using Lipofectamine 2000 (Life Technologies) according to the manufacturer's instructions. ON-TARGETplus non-targeting siRNA (herein referred to as si-scr, GE Healthcare) was used as a control.

HEK293T cells (American Type Culture Collection) were cultured and transfected as previously described (Hiepen et al., 2014).

### Expression plasmids

The full-length coding sequence of human VE-cadherin was amplified from HUVEC cDNA and cloned into the pcDNA3.1 mammalian expression vector using TOPO-TA cloning (Life Technologies) according to the manufacturer's instructions. The plasmids encoding BMPRII-HA and BMPRII-Myc were described previously (Gilboa et al., 2000). The C-terminally HA-tagged human ALK2 (ALK2-HA) expression plasmid was kindly provided by K. Miyazono (Tokyo, Japan). Full-length c-Src expression plasmid was kindly provided by R. Lefkowitz (Durham, USA) and is described elsewhere (Luttrell et al., 1999). All plasmids were verified by DNA sequencing.

### RNA extraction and qRT-PCR

HUVEC total RNA was isolated as previously described (Bhushan et al., 2013). Quantitative (q) RT-PCR was performed using SYBR Green Mix and the StepOne Plus Real-Time PCR System (Applied Biosystems). The following human-specific primer sets were used (5′-3′, forward and reverse): *ID1*, GCTGCTCTACGACATGAACG and CCAACTGAAGGTCCCTGATG; *CDH5*, CAGCCCAAAGTGTGTGAGAA and CGGTCAAACTGCCCATACTT; *GAPDH*, GAAGGTGAAGGTCGGAGTC and GAAGATGGTGATGGGATTTC. The ΔΔCt method was used to quantify mRNA expression relative to *GAPDH*.

### Immunoprecipitation and western blot analysis

Immunoprecipitation of endogenous proteins from HUVECs was performed using TNE lysis buffer freshly supplemented with 0.5% Triton X-100, 1 mM PMSF (Carl Roth), 20 mM sodium pyrophosphate, 50 mM sodium fluoride, 300 µM freshly prepared sodium pervanadate and 1× Complete EDTA-free Protease Inhibitor Cocktail (Roche). Immunoprecipitation of overexpressed proteins from HEK293T cells was performed using a modified radioimmunoprecipitation assay buffer (50 mM Tris-HCl pH 7.4, 150 mM NaCl, 0.5% NP-40, 0.1% SDS) freshly supplemented with inhibitors as described above. Following lysis, lysates were centrifuged at 4°C for 20 min at 16,000 ***g*** and pre-cleared at 4°C for 30 min with recombinant protein A-agarose beads (Thermo Scientific). Aliquots were then set aside for direct blot analysis [total cell lysates (TCLs)] and aliquots for immunoprecipitation (IP) were incubated at 4°C for 2 h or overnight with the indicated antibodies (5 μg/ml). Control samples were either incubated with isotype control antibodies or recombinant protein A-Sepharose beads (GE Healthcare). Immunocomplexes were precipitated at 4°C for 2 h with recombinant protein A-Sepharose beads (GE Healthcare) and subsequently washed three to five times with fresh lysis buffer including inhibitors. Proteins were eluted with 2× Laemmli sample buffer and heated for 10 min at 95°C.

Protein lysates were blotted and analyzed as previously described (Hiepen et al., 2014). Quantitation of signal intensities was performed using Bio1D software (Vilber Lourmat) or Image Studio Lite software (LI-COR Biosciences) and normalized to loading control or IP TCL and untreated control samples.

### Immunofluorescence microscopy

HUVECs were seeded at 200,000 cells/cm^2^ in HUVEC growth medium in µ-Slide 8-well (ibidi) dishes 24 h prior to the experiment. After 4 h of serum starvation in HUVEC starvation medium containing 1% FCS, cells were stimulated with the indicated growth factors at 37°C for 4 or 24 h. Subsequently, cells were fixed, quenched and permeabilized with 0.3% Triton X-100. After blocking, cells were incubated with the indicated primary antibodies (1:200), followed by detection using Alexa Fluor-coupled secondary antibodies (Life Technologies). Nuclei were counterstained using DAPI (Carl Roth). Images were taken with a CoolSnap-HQ2 CCD camera (Roper Scientific) mounted to an inverted epifluorescence Axiovert 200M microscope (Carl Zeiss) and analyzed with Axiovision (Carl Zeiss) and Corel DRAW X4 (Corel) software.

### Cell-surface biotinylation

The cell-surface biotinylation protocol is described by Gabriel et al. (2009). In brief, HUVECs were seeded at 20,000 cells/cm^2^ in HUVEC growth medium 48 h prior to the experiment, serum-starved in HUVEC starvation medium containing 2% FCS for 6 h at 37°C. Subsequently, cells were either stimulated with the indicated growth factors for 60 min at 37°C or left at 4°C for total cell-surface control samples. Biotinylation using cell-impermeable sulfosuccinimidyl-6-(biotinamido)hexanoate (EZ-Link Sulfo-NHS-SS-Biotin, Thermo Scientific) and subsequent processing were performed as previously described (Gabriel et al., 2009).

### Internalization assay

Internalization of VE-cadherin was measured as previously described (Xiao et al., 2003b). In brief, HUVECs were seeded at 50,000 cells/cm^2^ (sparse) or 200,000 cells/cm^2^ (confluent) in HUVEC growth medium in µ-Slide 8-well dishes 24 h prior to the experiment. After serum starvation, cell-surface VE-cadherin was labeled at 4°C with the extracellular VE-cadherin domain-targeting antibody BV6 for 60 min, rinsed with ice-cold PBS and stimulated with recombinant growth factors at 37°C. Then, cells were subjected to a mild acid wash and internalized VE-cadherin was visualized using immunofluorescence microscopy as described above. Internalized VE-cadherin vesicles from at least ten images per experimental condition were quantified using BlobFinder image analysis software as previously described (Allalou and Wählby, 2009). The results were normalized to untreated control cells.

### *In situ* proximity ligation assay

HUVECs were seeded at 200,000 cells/cm^2^ in HUVEC growth medium in Nunc Lab-Tec II 16-well glass chamber slides (Thermo Scientific). Cells were serum-starved for 2 h in HUVEC starvation medium, followed by stimulation with the indicated growth factors for 60 min. Subsequently, Duolink *in situ* proximity ligation (Sigma-Aldrich) was performed as previously described (Thymiakou and Episkopou, 2011). The number of heteromers was quantified using BlobFinder image analysis software as previously described (Allalou and Wählby, 2009). At least 15-20 images per experimental condition were quantified and normalized to untreated control cells.

### Transendothelial solute permeability

HUVEC monolayer solute permeability was determined using an *in vitro* vascular permeability 96-well assay kit (Merck Millipore) according to the manufacturer's instructions. HUVECs were stimulated with the indicated growth factors for 24 h and monolayer solute permeability was determined by addition of a high molecular weight FITC-Dextran (Merck Millipore) and subsequent measurement of fluorescence intensity using a Mithras microplate reader (Berthold Technologies). Fluorescence intensity was measured in duplicate per condition and normalized to untreated control cells.

### Transendothelial electrical resistance (TEER)

HUVECs were seeded at 200,000 cells/cm^2^ in HUVEC growth medium on fibronectin/collagen-coated Costar 24-well transwell inserts (0.4 µm polyester membrane, Corning) 72 h prior to the experiment. Cells were then serum-starved for 4 h in HUVEC starvation medium containing 2% FCS and stimulated with the indicated growth factors. TEER was measured at the indicated time points using a Millicell ERS-2 Voltohmmeter (Merck Millipore). Then, blank TEER values, measured in transwell inserts without cells, were subtracted, resistance per cm^2^ was calculated and normalized to untreated control cells at the start of stimulation (0 h).

### VE-cadherin declustering

Declustering of VE-cadherin was performed as previously described (Rudini et al., 2008). Antibodies were used at 50 µg/ml.

### Statistical analysis

Statistical analysis was performed by unpaired Student's *t*-test or variance analysis using SigmaPlot software (Systat Software). *P*<0.05 was considered statistically significant.
